# Extraintracranial Bypass as a Rescue Therapy for Symptomatic Flow Diverter Thrombosis

**DOI:** 10.1155/2015/204387

**Published:** 2015-09-17

**Authors:** Luigi A. Lanterna, Alessandro Lunghi, Carlo Brembilla, Paolo Gritti, Claudio Bernucci

**Affiliations:** ^1^Department of Neuroscience and Surgery of the Nervous System, Papa Giovanni XXIII Hospital, 24127 Bergamo, Italy; ^2^Department of Neuroradiology, Alessandro Manzoni Hospital, Lecco, Italy; ^3^Department of Anesthesiology, Papa Giovanni XXIII Hospital, 24127 Bergamo, Italy

## Abstract

A 56-year-old female with a giant partially thrombosed unruptured carotid-ophthalmic aneurysm was treated with a Pipeline flow diverter. Three months after the procedure, in concomitance with the discontinuation of one of the antiplatelet medications, the patient suffered from a minor stroke and relapsing transient ischemic attacks. The angiography demonstrated the occlusion of the internal carotid artery, and a perfusion-weighted CT scan showed a condition of hypoperfusion. The patient underwent a double-barrel extraintracranial bypass. The postoperative course was uneventful and she has experienced no further ischemic events to date.

## 1. Introduction

There is increasing evidence that the flow diverters (FDs) are changing the classical treatment paradigm of cerebral aneurysms, from the occlusion of the sac to the reconstruction and healing of the vessel wall [1]. The intention of the FD is to trigger aneurysm thrombosis by inducing a modification of the haemodynamics inside of the aneurysm. Although the use of FDs is still in its infancy and the flow diversion in aneurysms trial (FIAT) is still ongoing [2], recent data suggest that the procedure is technically feasible with an acceptable effectiveness profile in selected cases [1, 3]. However, despite the appeal that derives from the opportunity to overcome the limitations of the classical coiling and the invasiveness of surgery, the procedure has some drawbacks and a nonnegligible rate of thrombotic or haemorrhagic complications with a combined fatality and disability rate of about 10% [1, 4, 5].

We describe the case of a patient who was treated with a FD for a giant internal carotid artery (ICA) aneurysm in whom an extraintracranial (EC-IC) bypass reversed a condition of ongoing cerebral ischemia caused by the FD thrombosis and consequent occlusion of the ICA.

## 2. Case Report

**Figure 1:**
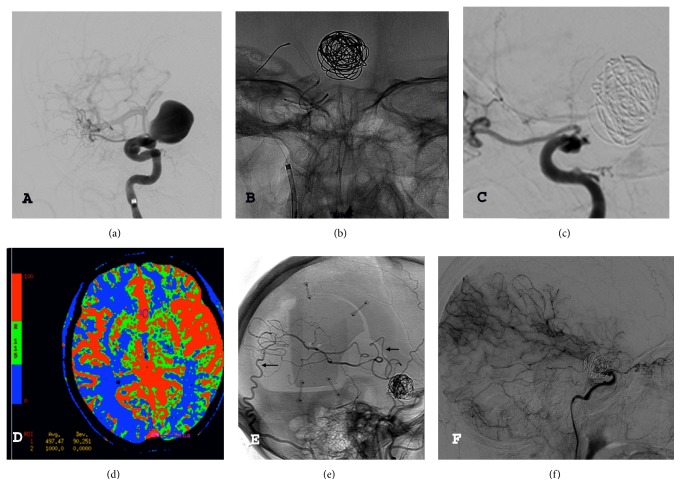
(a) Digital subtraction angiography showing a giant carotid-ophthalmic aneurysm on the right side. (b) Coiling and flow diverter deployment. (c) Digital subtraction angiography showing the occlusion of the internal carotid artery on the right side. (d) Perfusion-weighted CT scan: reduced cerebral blood flow on the right hemisphere. (e-f) Postoperative digital subtraction angiography (early and late phases) showing the patency of the double-barrel bypass (arrows) and the territory perfused by the bypass.

**Figure 2:**
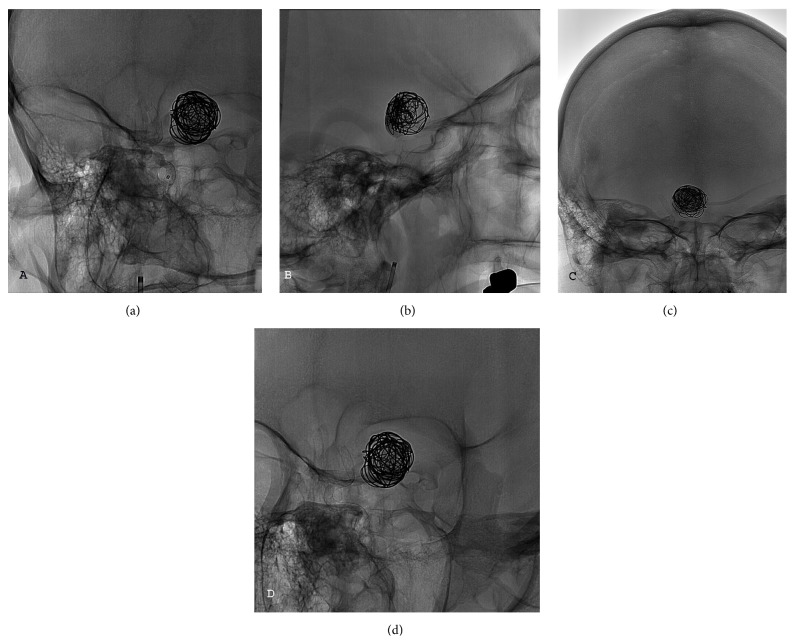
(a to d) The images show the FD in different projections.

A 56-year-old female was initially admitted to the neurosurgical department because of retroorbital pain and mild visual deterioration in both eyes. The ophthalmologic examination showed a bilateral constriction of the visual field and a slight reduction in the visual acuity on the right side. A magnetic resonance of the brain and a digital subtraction angiography (DSA) disclosed a giant partially thrombosed aneurysm of the ICA on the right side (Figure 1(a)). Although there is no randomized evidence showing that the FDs are superior to the conventional treatment options, the FD option was considered for the following reasons: the paraclinoid ICA represents one of the best targets for such devices [6], there were no anatomical limitations to a correct deployment, and, by inducing aneurysm shrinkage, it might have been possible to relieve the mass effect on the optic pathways [7]. The ophthalmic aneurysm was initially loosely coiled and then a Pipeline FD was placed in the parent artery across the aneurysm neck (Figure 1(b)). The FD was well deployed and the landing zone extended from the supraclinoid ICA to the cavernous ICA (Figure 2). There was only a mild focal narrowing at the level of the distal ring where the ICA was compressed by the bulk of the aneurysms against the clinoid process (Figure 2). The visual acuity deteriorated acutely on both sides despite corticosteroids. The patient was discharged home after 7 days with double antiplatelet medication (75 mg of clopidogrel and 100 mg of aspirin per day). Three months after the procedure, in concomitance with the cessation of the clopidogrel, she acutely developed a hemiparesis on the left side that lasted 48 hours. Then, she began suffering from transient ischemic attacks (TIAs). A DSA disclosed the thrombosis of the FD with occlusion of the ICA (Figure 1(c)). The patient continued suffering from multiple TIAs despite the resumption of antiplatelet medications, adequate hydration, and a thorough blood pressure monitoring to prevent hypotension. A perfusion-weighted CT scan demonstrated a significant increase in the mean transit time and a reduction in the cerebral blood flow on the right side (Figure 1(d)). The patient underwent a double-barrel extraintracranial (EC-IC) bypass with the occipital artery and the parietal branch of the superficial temporal artery as donors. Her postoperative course was uneventful and she has experienced no further TIAs. The follow-up DSA 3 months later confirmed the patency of the bypasses (Figures 1(e)-1(f)).

## 3. Discussion

This report describes a condition where an EC-IC bypass was performed to reverse a condition of ongoing cerebral ischemia and a risk of impending stroke related to the thrombosis of a FD and parent artery occlusion (PAO). Until now, apart from a patient who underwent open surgical bailout with STA-MCA bypass and intentional ICA sacrifice to relieve optic chiasm compression after unsuccessful treatment of a giant ICA aneurysm with a FD [8], no patient with symptomatic FD thrombosis who could benefit from an EC-IC bypass has been described. This case shows that, in addition to patients with acute and large brain infarctions for whom there are only limited therapeutic opportunities, there could be patients with still salvageable brains who are at a high risk of major stroke or stroke evolution and who could benefit from prompt surgical revascularization procedures.

The FD represents the most significant evolution of the endovascular approach for cerebral aneurysms. Although this device is undoubtedly appealing as it overcomes some of the limitations of the conventional endovascular procedures, this approach is not without drawbacks. A recent multicentric retrospective study has reported a mortality rate of 5.9% and a morbidity rate of 3.7% for a series of unruptured aneurysms [4]. Recent systematic reviews of the literature found a mortality rate of 3.5–4% and a morbidity rate of 5–6.2% [4, 5]. Similarly, Grossberg and colleagues [9] revealed a combined mortality and morbidity rate of about 6.5% in a series of patients with unruptured carotid-ophthalmic aneurysms. Although some of the poor results may be attributed to bleeding or worsening of the mass effect of the aneurysm, a significant proportion of the unfavourable results were related to ischemic events that may derive from PAO, whose overall incidence varies from 0% to 10% [10–13].

Intrastent thrombosis and PAO, though not always clinically symptomatic, account for a significant part of the mortality and morbidity rates imputable to cerebral ischemia. Berge et al. [10] reported 6 PAOs and in 4 of them (67%) PAO resulted in stroke. Piano et al. [12] described 5 PAOs, one occurring acutely within the first 24 hours after the procedure and 4 being delayed. The acute PAO was symptomatic and the patient died. Szikora et al. [13] observed 1 PAO in 19 procedures and it was symptomatic for a minor stroke. Likewise, Byrne et al. [11] reported that, in one out of the three patients with delayed permanent neurological worsening, the cause was a PAO-related stroke. Although the literature still remains sparse and sometimes contradictory, FD thrombosis with PAO appears to be one of the most clinically significant complications of FDs.

It should be pointed out that, as in our patient, FD thrombosis may happen even several months after deployment, and it may be related to the discontinuation of the antiplatelet therapy. While, on the one hand, this observation emphasises the importance of long-term treatment with antiplatelet medications, on the other hand, it suggests a possible drawback that may affect the long-term risk profile of the FDs. In particular, recent data regarding patients with myocardial infarction have shown that adherence to antiplatelet medications may change during long-term therapy and problems with compliance are not uncommon [14]. The ongoing FIAT [2] that compares the risk and effectiveness of FDs with conventional therapies could provide an answer to the question as to whether or not the drawbacks of FDs may outweigh their theoretical benefits and, therefore, provide evidence for the clinical decision-making process.

The pathogenesis of cerebral ischemia in PAO may be thromboembolic or haemodynamic or a combination of the two. At the present time, in acute PAOs with thrombosis of vital perforating arteries or massive thromboembolic events, there are limited treatment options apart from medical therapy. A completely different condition occurs when the ischemic symptoms are, at least in part, of haemodynamic origin as in our patient. The recent COSS trial [15] and the International EC/IC trial [16] investigating the preventive effectiveness of bypass failed to find a benefit from the procedure. However, these studies included only chronic patients who had already passed through the acute phase, when the risk of stroke recurrence or evolution is higher. The results of the trials are, therefore, not applicable to cases of acute symptomatic occlusion as in our patient or, in general, to those who suffer from acute FD thrombosis. Patients with acute haemodynamic insufficiency fare poorly and the cerebral bypass may be a valuable, safe, and effective option [17].

In conclusion, one of the possible complications related to the use of the FDs is cerebral ischemia caused by FD thrombosis and PAO. In a subgroup of patients with PAO the cerebral ischemia may be of haemodynamic origin. It is important to promptly identify this subgroup of patients as an EC-IC bypass may prevent stroke-related neurological deterioration.
